# Transmission of SARS-Cov-2 and other enveloped viruses to the environment through protective gear: a brief review

**DOI:** 10.1007/s41207-021-00251-w

**Published:** 2021-04-07

**Authors:** Francesco Petrosino, Debolina Mukherjee, Gerardo Coppola, Maria Teresa Gaudio, Stefano Curcio, Vincenza Calabro, Francesco Marra, Prosun Bhattacharya, Umapada Pal, Nabil Khélifi, Sudip Chakraborty

**Affiliations:** 1 Laboratory of Transport Phenomena and Biotechnology, Department of D.I.M.E.S, University of Calabria, Via- P. Bucci, Cubo-42a, 87036 Rende, CS Italy; 2 Department of Industrial Engineering, University of Salerno, Via Giovanni Paolo II, 132, 84084 Fisciano, SA Italy; 3 Department of Sustainable Development, Environmental Science and Engineering, KTH Royal Institute of Technology, Teknikringen 10B, 10044 Stockholm, Sweden; 4 Instituto de Física, Benemérita Universidad Autónoma de Puebla, Apdo. Postal J-48, 72570 Puebla, Mexico; 5 Springer, a Part of Springer Nature, Tiergartenstrasse 17, 69121 Heidelberg, Germany

**Keywords:** Enveloped virus, Virus transmission, Virulence factor, Personal protective equipment (PPE)

## Abstract

Over the past two decades, several deadly viral epidemics have emerged, which have placed humanity in danger. Previous investigations have suggested that viral diseases can spread through contaminants or contaminated surfaces. The transmission of viruses via polluted surfaces relies upon their capacity to maintain their infectivity while they are in the environment. Here, a range of materials that are widely used to manufacture personal protective equipment (PPE) are summarized, as these offer effective disinfection solutions and are the environmental variables that influence virus survival. Infection modes and prevention as well as disinfection and PPE disposal strategies are discussed. A coronavirus-like enveloped virus can live in the environment after being discharged from a host organism until it infects another healthy individual. Transmission of enveloped viruses such as SARS-CoV-2 can occur even without direct contact, although detailed knowledge of airborne routes and other indirect transmission paths is still lacking. Ground transmission of viruses is also possible via wastewater discharges. While enveloped viruses can contaminate potable water and wastewater through human excretions such as feces and droplets, careless PPE disposal can also lead to their transmission into our environment. This paper also highlights the possibility that viruses can be transmitted into the environment from PPE kits used by healthcare and emergency service personnel. A simulation-based approach was developed to understand the transport mechanism for coronavirus and similar enveloped viruses in the environment through porous media, and preliminary results from this model are presented here. Those results indicate that viruses can move through porous soil and eventually contaminate groundwater. This paper therefore underlines the importance of proper PPE disposal by healthcare workers in the Mediterranean region and around the world.

## Introduction

During a viral epidemic, personal protective equipment (PPE) plays a major role in protecting doctors, nurses, and other healthcare or emergency medical personnel from viral infections (Islam 2020). PPE is protective gear that has been fabricated from nonporous impermeable materials such as plastics to maintain a strategic distance between the PPE wearer and substances potentially contaminated with infectious viruses. This is the lowest level of equipment for preventing infections that can be used by any healthcare worker. However, the efficiency of a PPE kit depends on how carefully it is used (Phan et al. 2018). It is especially important to utilize PPE when a viable immunization or antiviral/antimicrobial vaccine is not yet available. Wearing PPE helps healthcare professionals and members of other specialist organizations to feel safe and to work successfully during pandemics (Andersen 2019).

To be transported into the environment, pathogens must be able to stay viable outside the host. How long a pathogen remains viable in the environment depends on the effects of numerous biotic and abiotic stresses on the pathogen (Wolff et al. 2005). Viral infections such as COVID-19 spread from an infected person to a healthy person mainly through direct contact or aerosol generation, which indicates that environmental factors play a significant role in the spread of viral diseases. Environmental factors such as air humidity, ambient temperature, and polluted surfaces are critical influences on virus survival and subsequent transmission (Prussin et al. 2018). Healthcare workers must change their protective equipment after a certain period of time, and such equipment must be decontaminated using recommended standard procedures before being reused (Islam 2020; Phan et al. 2018; CDC 2020a). Personal protective equipment should be worn in the manner specified in the guidelines of the World Health Organization (WHO) and decontaminated after use so that enveloped virus (SARS-CoV-2) on the outer surface is not transmitted to healthy individuals (Islam 2020). Hand sanitization is essential before and after using PPE, and a suitable protocol for wearing and removing it should be followed; not following such a protocol could lead to a high probability of virus transmission via PPE (Feng et al. 2020; CDC 2020b). Due to the threat posed by infectious waste in hospitals and other emergency healthcare clinics, access to medical waste should not be granted without the permission of medical professionals. According to the WHO protocol, all PPE must be transferred safely to a sterilization facility for decontamination.

Hazardous healthcare waste normally comprises about 10% of the waste from healthcare facilities worldwide (World Health Organization 2014); however, during a pandemic, this proportion increases drastically. Also, while used PPE kits are not normally considered highly infectious waste (in contrast to waste contaminated with blood or other body fluids and laboratory cultures and stocks), they are considered highly infectious during pandemics. Therefore the collection, handling, transport, treatment, and disposal of PPE kits during a pandemic need special attention.

A national policy on healthcare waste disposal should allow for regional differences and variations in local capacity and socioeconomic conditions. International guidelines in this regard are available in the documents produced by the WHO, the United Nations Environment Programme—Secretariat of the Basel Convention, and several nongovernmental organizations (NGOs) (e.g., WHO 2005). Among the international treaties, the Stockholm Convention is a notable global treaty to protect human health and the environment from persistent organic pollutants (POPs). The Stockholm Convention states that “priority consideration” should be given to alternative processes with a similar usefulness but that avoid the formation and release of organic pollutants. The best available technique (BAT) or best environmental practice (BEP) guidelines describe alternative technologies such as steam sterilization, advanced steam sterilization, microwave treatment, dry-heat sterilization, alkaline hydrolysis, and biological treatment (UNEP 2006) for the disinfection of healthcare wastes in general (World Health Organization 2014). It should be noted that the treaties, policies, and guidelines (national and international) framed by different countries and international bodies are general protocols for the management of healthcare wastes; they are not specific to PPE. Until now, only 24% of the countries around the world have adopted dedicated healthcare legislation on waste management. However, very recently (after the COVID-19 pandemic started in 2019), the Institute of Global Environmental Strategies (IGES) and the WHO jointly published a fine report entitled* Waste Management During the COVID-19 Pandemic: From Response to Recovery* under the United Nations Environment Programme (2020), in which they provide clear instructions for health workers on the proper use of PPE (collection, transfer station, informal sector, etc.). The report also focuses on the management of healthcare waste during the COVID-19 pandemic, covering healthcare waste generated by hospitals, medical centers, and emergency medical facilities as well as municipal solid waste (MSW).

Although there are no specific and well-planned strategies for PPE disposal and reutilization at the national or international level, the severity and devastating nature of COVID-19 have prompted governments and governmental statutory bodies in several countries to formulate contingency plans. For example, the CPCB (Central Pollution Control Board) of India states that used PPE (such as face shields, goggles, hazmat suits, plastic coveralls, masks, head covers, and shoe covers) from COVID-19 isolation wards at healthcare facilities should be segregated and sent to common facilities for disposal as per biomedical waste management rules (BMWM rules). However, used PPE (such as masks and gloves) from households, commercial establishments, and institutions must be stored separately for a minimum of 72 h, cut and shredded, and then disposed along with solid waste (Times of India 2020). Similarly, the European Centre for Disease Prevention and Control (ECDC) has provided guidelines for the selection, handling, and disposal of PPE kits (ECDC 2020). The US Food and Drug Administration (FDA) provides clear guidelines on the selection, utilization, and reutilization of PPE (FDA 2020), and the Army Public Health Center (APHC) has published lucid guidelines on the disposal of PPE kits for workers caring for COVID-19 and non-COVID-19 patients that follow the FDA guidelines (APHC 2020).

Many countries do not have the proper facilities to treat contaminated PPE kits, so they are dumped on landfills. This was one of the motivations for the present paper, which reviews various aspects of PPE usage and enveloped virus transmission and reports a modeling study of the diffusion of the coronavirus through a porous medium (soil here) via the leaching of contaminated water.

## PPE and enveloped virus transmission

Personal protective equipment should include eye and face protection, hand protection (disposable gloves), respiratory protection (an N95 mask or equivalent), skin and body protection, head protection, and reusable leather boots (Mahmood et al. 2020). In Table 1, we list materials that are commonly utilized to manufacture PPE components. If any of these PPE components comes into contact with an infected patient, the entire PPE kit should be discarded in a scientific way, as instructed by the local regulatory authority. Therefore, every component of a single-use PPE kit is selected with contamination and safety control in mind. Gloves are the most commonly discarded PPE component utilized by healthcare professionals around the world, as they are strictly for single use. They should be changed after direct contact with an infected patient, otherwise the gloves could help the disease to spread (Loveday et al. 2014). Other components of a PPE kit can be reused after appropriate decontamination. PPE sterilization has become an economic and useful step due to the increased demand for such components during the COVID-19 epidemic. The general sterilization procedure involves autoclaving the components at 121 °C for 15 min (Wilson and Nayak 2013). PPE components such as skin protectors (gowns), shoes, and face protectors (masks) can easily be decontaminated using a neutral detergent or disinfectant such as 0.5% chlorine solution (Rutala and Weber 2016). However, there are some disadvantages of reusing PPE; for instance, heat-resistant viruses may remain active after thermal sterilization (Riley et al. 2017).

**Table 1:** Materials used in different PPE components

PPE component	Raw material used
N95 respirators	Polypropylene
Powered air-purifying respirators	Rubber or silicone
Face shields	Polycarbonate, propionate, acetate, polyvinyl chloride, and polyethylene terephthalate glycol
Normal surgical masks	Polypropylene
Goggles	High-quality polycarbonates
Single-use protective gowns	(Normally) polypropylene
Coveralls	High-density polyethylene

## Disposal of PPE kits

Personal protective equipment must be free from any kind of contaminant, as any kind of infectious substance on the PPE could be transmitted to and harm a healthy individual (Mahmood et al. 2020; IRIS 2020). All the components of single-use PPE should be treated appropriately, whereas reusable PPE should be sterilized before reutilization according to an appropriate decontamination procedure (Islam 2020; Phan et al. 2018; CDC 2020a). However, it is important to consider whether the decontamination process will cause the PPE to lose its protective ability. When properly disposing of single-use PPE, it should be safely stored by the hospital or laboratory in a separate supervised area. PPE components such as masks and eye and skin protectors can be reused after any of the following decontamination processes:

- Protective masks such as N95 can be sterilized by applying 7.5% hydrogen peroxide solution or 0.2% peracetic acid solution for 8–45 min at 20 °C. However, this disinfection process can reduce the efficacy of masks by blocking the pores in their internal layers. In such cases, the respirator must be replaced (Chakraborty et al. 2020).
- The clothes can be dipped in 70% ethyl alcohol solution or 100 ppm chlorine solution for more than 1 min (Lamptey 2020). Clothes should be checked for holes before sterilization; if any hole is found, the clothing must be discarded according to the hospital guidelines.
- Continuous washing using detergent is the method most commonly used to decontaminate leather boots. After detergent treatment, the boots should be dried quickly in sterilized warm air to reduce cross-contamination (Mahmood et al. 2020).
- Goggles can be reutilized after wiping them with a disinfectant such as 60–90% ethanol or 2–4% aqueous chlorhexidine. Alcohol-based rubbing is the most basic and effective sterilization process for eye protectors; it is even more effective than detergent solutions. However, as a precautionary measure, the healthcare worker should sanitize their hands before wiping the goggles (Boyce and Pittet 2002).

Vapor-phase hydrogen peroxide (VPHP) treatment, thermal disinfection (heat) treatment, ultraviolet germicidal irradiation (UVGI), ethylene oxide (EtO), and bleaching (sodium hypochlorite) treatment are generally used to systematically disinfect PPE. Among these, UVGI and EtO treatment effectively decontaminate PPE components without affecting their protective performance or physical appearance. On the other hand, a single cycle of VPHP treatment does not have a significant effect on respirator performance or appearance. While bleaching (0.1% sodium hypochlorite) treatment does not affect the appearance or performance of the treated respirator, the residual odor of bleach following the treatment is a potential health risk. In fact, even the presence of a low concentration of this chemical led researchers to discourage the use of bleach for decontamination. The effectiveness of common decontamination techniques has been nicely reviewed by Kharbat et al. (2020).

## Factors affecting virus survival rates

**Fig. 1:**
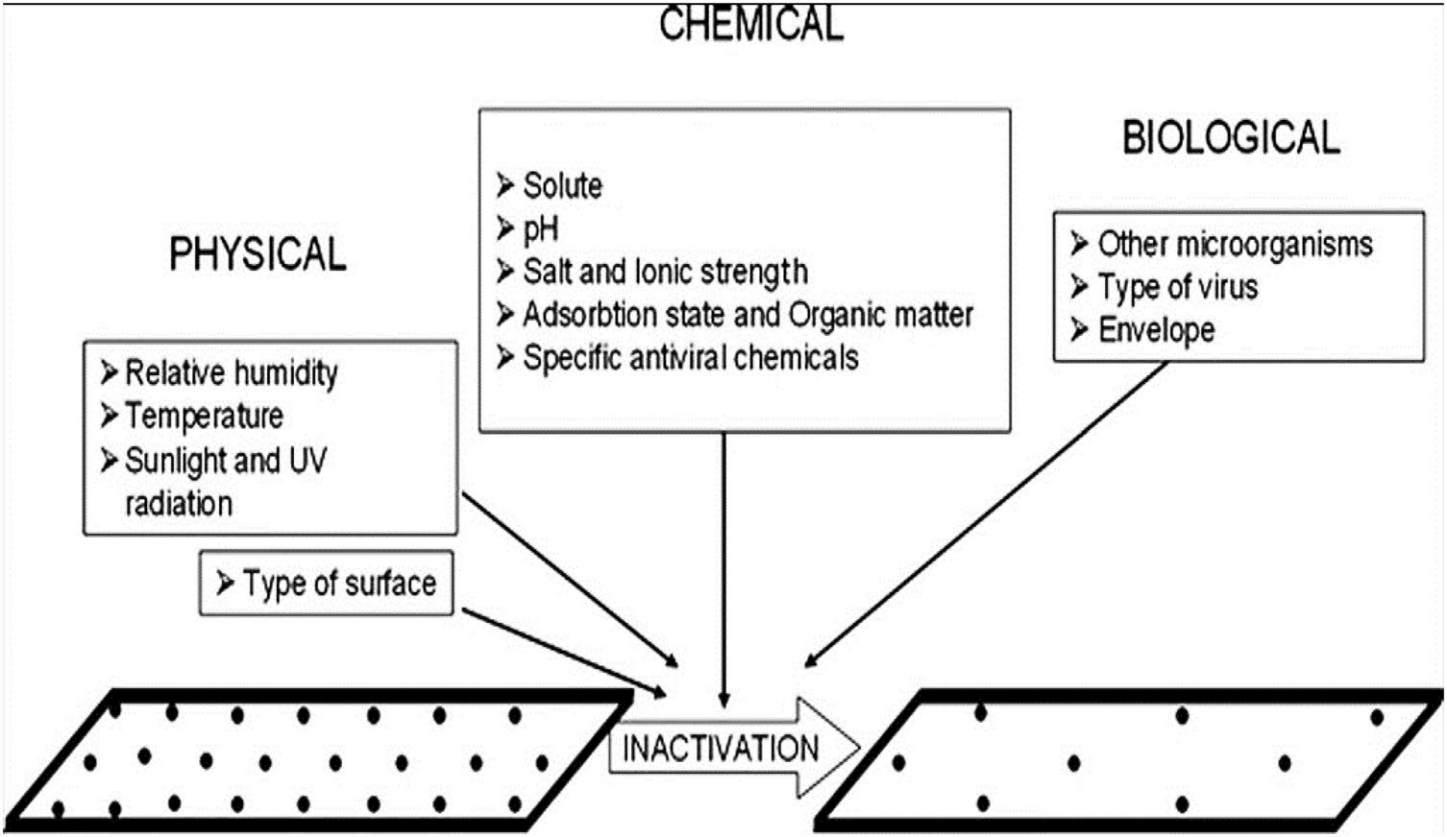
Factors that affect virus survival in the environment

There are several factors that affect the ability of enveloped virus (SARS-CoV-2) to retain its infectivity for a certain period of time on a surface. Environmental factors influence this ability in different ways, with each having some favorable and unfavorable effects. Physical factors include the ambient humidity, temperature, and sunlight (Kim et al. 2018; Casanova et al. 2010a, b; Lin and Marr 2020). There are also some chemical and biological factors (Ye et al. 2018) that affect the viability of a virus on a surface (Fig. 1). While some environmental factors aid virus transmission, a few suppress the spread of enveloped virus (SARS-CoV-2) to healthy individuals.

## Virus transmission modes

Infectious viruses can be spontaneously transmitted from one person to another in several ways. In the particular case of SARS-CoV-2, infected people who are asymptomatic are more likely to spread the infection (Robel et al. 2020). Enveloped virus (SARS-CoV-2) can be transmitted between people in relatively close contact, but it can also remain viable on a surface after being discharged from the host organism. If a healthy individual stays in the same room as an infected person, it is possible that she/he can be infected through air movement. However, not all viruses present airborne transmission (e.g., HCV and HIV do not), and the potential for airborne contamination with SARS-CoV-2 and enveloped viruses is yet to be confirmed (Andersen 2019; Wolff et al. 2005). Examples of enveloped viruses include ebola, avian flu virus, Zika, MERS, and the recently discovered SARS-CoV-2 coronavirus. Recent studies have revealed that enveloped viruses have a lipid bilayer on their cell walls and they can mutate over time. These structural properties help enveloped viruses to remain stable when they are under stress (Krista and Alexandria 2020). Coronaviruses can be transmitted through the air, via droplets, by direct contact with an infected person, and through fecal matter (Cai et al. 2020; van Doremalen et al. 2020).

Transmission through an aerosol is the most common way of spreading enveloped viruses. An aerosol is a suspension of small water droplets generated by sneezing and talking (Nicas et al. 2005; Cook 2020; Chan et al. 2004; Yu et al. 2004). Such water droplets have an average diameter of less than 5 µm. Infected droplets can travel a small distance (less than 2 m) through the air. After entering the air, gravity causes enveloped virus (SARS-CoV-2) to settle and land on a surface (van Doremalen et al. 2020). However, smaller droplets can travel further than bigger ones. Whereas bigger droplets take less time to settle under the influence of gravity, smaller droplets tend to remain in the air for a longer period of time. Air movement greatly affects the settling time of enveloped virus (SARS-CoV-2). Air turbulence is directly proportional to the time taken for enveloped virus (SARS-CoV-2) to settle down (Nicas et al. 2005). If the droplet diameter is less than 10 µm, enveloped virus (SARS-CoV-2) reaches the lungs directly through the respiratory tract. To prevent disease transmission through aerosols, social distancing should be maintained, and gloves, an N95 face mask, eye protection, and a flower-sleeve gown should be worn by healthcare workers (Cook 2020). Viral transmission can happen through direct contact with an infected patient’s body liquids or through contact with fomites (tainted inanimate items and surfaces, e.g., the floor, utensils, and bedclothes that have recently been contaminated with body liquids) (Stowell et al. 2012). Disease can be transmitted through broken skin and wounds (e.g., needlestick injuries). Previous studies have demonstrated that most of the Middle East Respiratory Syndrome Coronavirus (MERS-CoV) viruses remain viable in adults after 60 min of aerosolization (Islam 2020; Oran and Topol 2020). Enveloped virus (SARS-CoV-2) can be transmitted through stool, mucus, and serum but is usually less stable in the urine. Contaminated stools represent a danger to human health, but they can be washed away with water. Fecal transmission of enveloped virus (SARS-CoV-2) is possible because enveloped virus (SARS-CoV-2) can survive in the human gastrointestinal (GI) tract before entering the environment in human stools (Oran and Topol 2020). Virus transmission can also occur indirectly via inanimate substances such as polluted surfaces, clothes, and materials. Enveloped viruses have the ability to resist abiotic and biotic environmental stress and survive on open surfaces, infecting healthy individuals through the nose, mouth, and eyes. The survival time of viruses on porous surfaces is shorter than that on nonporous surfaces (more than 72 h) (Phan et al. 2019).

## Preventive measures and PPE disposal

The PPE sterilization procedures discussed above should be followed to maintain a hygienic environment and prevent virus transmission. Hydrogen peroxide has been used in conjunction with steam to disinfect porous materials such as masks. It is a reliable disinfectant that is widely used worldwide because it does not block the pores of masks and does not damage their inner layers. Ethyl alcohol is another effective sanitizing agent and virucide. 70% ethyl alcohol is used to disinfect PPE clothing and eye protectors, as it kills or destroys more than 90% of microorganisms, including viruses and bacteria. So, after washing them with ethyl alcohol, PPE components are completely free of contamination. Disinfection methods are currently particularly important due to shortages of PPE (Mahmood et al. 2020). The best way to overcome this shortage is to regularly decontaminate the PPE that is available so that it can be reused. Disinfecting PPE kits instead of discarding them after a single use is also financially prudent, as these kits are expensive to produce and therefore purchase. As per hospital guidelines, other infectious clinical waste should be discarded in a separate place.

PPE kits that are in very poor condition should be disposed of in a separate container. Kit components such as damaged gowns, defective masks, and blood-stained materials are considered to be in poor condition and should not be reused. Some of the basic precautionary steps that should be implemented to deal with defective PPE (Krista and Alexandria 2020) are summarized in Table 2.

**Table 2:** Some precautionary steps that should be followed during the disposal of PPE kits (Robel et al. 2020)

1. Select an appropriate container to dispose of the PPE
2. Check the capacity and disposal time of the container
3. The container should be labeled with its maximum carrying capacity and the risks associated with it
4. To increase the carrying capacity of the container, a compression tool should be installed
5. The same equipment should not be reused for another container
6. The container must be placed in an isolated area to reduce cross-contamination

People involved in the disposal process should also wear PPE kit correctly. All the appropriate precautionary steps must be taken by healthcare organizations and their workers to avoid the dangerous consequences of exposure to viral diseases. Only medical professionals should be allowed to have direct contact with patients. According to medical experts, while these practices do not guarantee complete prevention of viral infections, they can prevent early transmission of enveloped virus (SARS-CoV-2). Regardless of the current situation, the emergency procedures recommended by the Centers for Disease Control and Prevention (CDC) should be followed (Phan et al. 2019; Rohrer et al. 2020; Lessler et al. 2009). The current epidemic (COVID-19) is thought to be transmitted through direct contact and airborne routes, and has been spreading from individual to individual for the last 12 months. The SARS-CoV-2 virus is considered to be more dangerous than other SARS viruses because it can remain at an asymptomatic stage in a healthy person. At this stage, the person does not show any symptoms of the disease but they can still transmit enveloped virus (SARS-CoV-2). Therefore, all the precautionary measures discussed earlier should be followed during this viral epidemic.

If disinfection is not performed properly, viruses attached to the PPE surface can become contagious and infect healthy people. On the PPE surface, enveloped virus (SARS-CoV-2) is subject to many environmental stresses but retains its infectivity (Chan et al. 2004). The humidity of the air is a major influence on virus survival. If the relative humidity is 50%, enveloped virus (SARS-CoV-2) can survive well and replicate freely. As the relative humidity increases, the enveloped virus survival rate decreases. Enveloped viruses have lipid bilayers in their cell walls, and this unique property helps them to survive at low humidity levels. The environmental temperature also affects virus survival on the PPE surface. High temperatures are harmful to enveloped virus (SARS-CoV-2), while low temperatures can help enveloped virus (SARS-CoV-2) to remain viable for longer (1–2 days). Sunlight is the main source of heat (elevated temperature) in the environment. The UV radiation in sunlight kills some viruses, but enveloped viruses can survive exposure to UV radiation and daylight due to their lipid bilayers. However, enveloped virus (SARS-CoV-2) survives best in darkness rather than daylight (Sagripanti and Lytle 2020; Casanova et al. 2010c).

## Factors affecting virus transmission

**Fig. 2:**
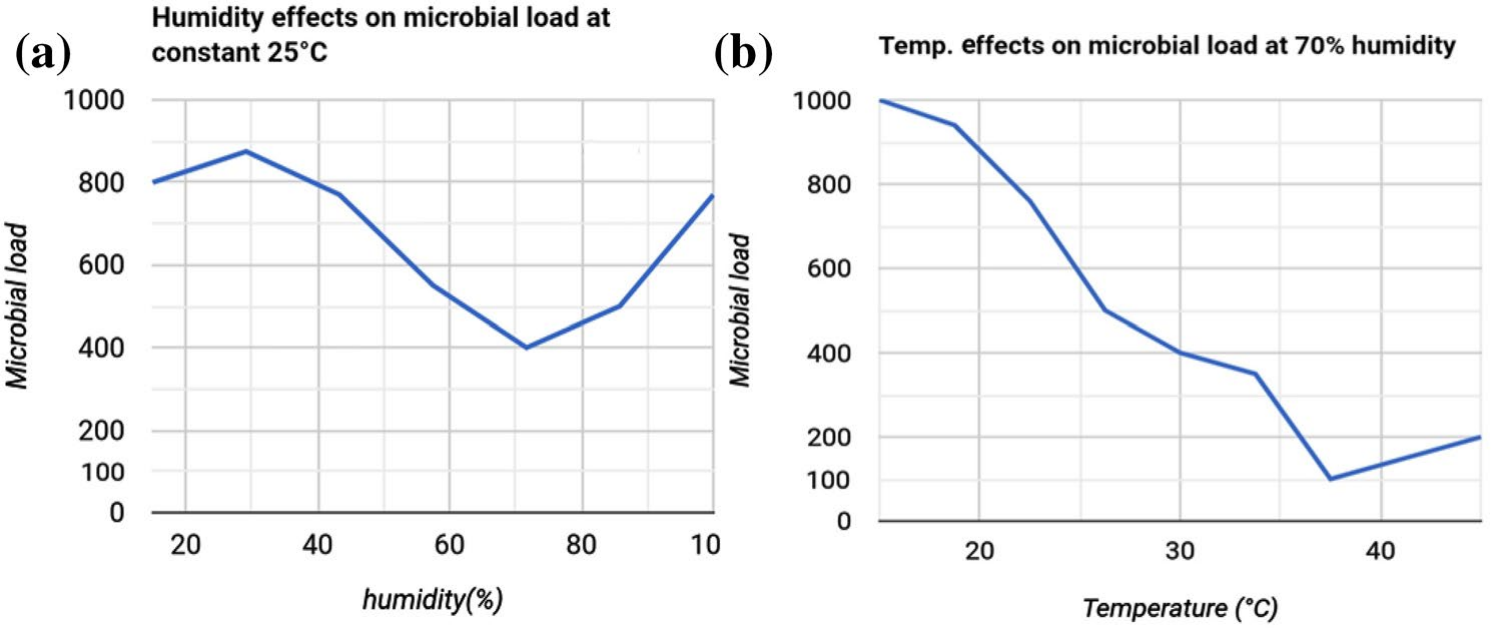
Effects of** a** ambient humidity and **b** temperature on virus survival rate (Suman et al. 2020)

Several factors are responsible for not only the transmission of viruses but also their deactivation. Figure 2 shows the relationship between virus inactivation and humidity (%). At constant temperature, enveloped virus (SARS-CoV-2) survival rate increases when the relative humidity is less than 50% or more than 90%. Increasing the relative humidity from 60% to 80% causes enveloped virus (SARS-CoV-2) survival rate to gradually decrease (Prussin et al. 2018). As the humidity increases, enveloped virus (SARS-CoV-2) remains stable in respiratory droplets for longer, so the rate of infection does not decrease considerably. As the relative humidity increases from 60% to 70%, the rate of viral infection via aerosols and droplets gradually increases (Stowell et al. 2012). As can be seen in Fig. 2b, for a specific relative humidity, the rate of virus inactivation gradually decreases with increasing temperature. The viral load and the infectivity of enveloped virus (SARS-CoV-2) are higher at low temperatures, such as between 15 and 20 °C, than at higher temperatures. Virus infectivity decreases with temperature, as increasing the temperature has a negative effect on virus survivability (Wolde 2020).

In closed environments, pH has only a minimal effect on enveloped virus (SARS-CoV-2). Generally, enveloped viruses prefer low-pH environments. An alkaline environment is harmful to enveloped virus (SARS-CoV-2) (Phan et al. 2019). The human stomach and gastrointestinal (GI) tract are acidic, which helps enveloped virus (SARS-CoV-2) to replicate in these organs. After replicating in large quantities, enveloped virus (SARS-CoV-2) passes out of the body through the feces and contaminates the environment. Contaminated surfaces play a role in disease transmission. Enveloped virus (SARS-CoV-2) can live on contaminated surfaces, and they can be transferred from those surfaces onto hands or other protective clothing, spreading infection. If the adsorption rate of the surface is high, enveloped virus (SARS-CoV-2) will remain viable there for a longer period of time (a week or, in some cases, even a month) (Lessler et al. 2009). Thus, the adsorption capacity of the surface is directly proportional to enveloped virus (SARS-CoV-2) survival rate. The existence of a microbial population negatively affects enveloped virus (SARS-CoV-2) survival rate. If viruses and microbial populations are present on the same surface they compete for nutrients, so enveloped virus (SARS-CoV-2) replication rate becomes very low (Stowell et al. 2012). Other factors that promote viral inactivation include the presence of disinfectants, chlorine, a high pH, particular surface properties, and air circulation. Data on the effects of these factors on virus viability are extremely helpful when planning and implementing appropriate steps for controlling and preventing viral diseases. In fact, if at all possible, controlling these environmental factors is an excellent alternative approach to controlling viral infections.

**Fig. 3:**
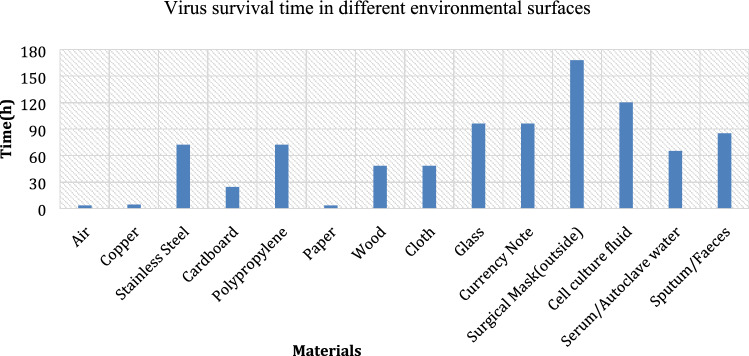
SARS-CoV-2 survival times on different environmental surfaces (Suman et al. 2020)

As different surfaces exert different environmental stresses, viruses are not equally as viable on all surfaces. As shown in Fig. 3, coronavirus can survive on a variety of environmental surfaces for periods ranging from several minutes to several days. The survival rate of enveloped virus (SARS-CoV-2) is highest in culture media: it can be transferred from culture media after 4–5 days. Most cultures are maintained at low temperatures, and the presence of abundant nutrients helps enveloped virus (SARS-CoV-2) to replicate and survive. Autoclave water is free from microbial populations, and enveloped virus (SARS-CoV-2) survival time in autoclave water is shorter than that in culture media due to the scarcity of nutrients in the water. Enveloped viruses can remain viable in feces for up to 4 days and can be washed off with water. However, enveloped virus (SARS-CoV-2) is less stable in the urine and has a survival time of less than 6 h (Suman et al. 2020; Sagripanti and Lytle 2020; Casanova et al. 2010c; Selcuk et al. 2021; Al-Kindi et al. 2020). Enveloped virus (SARS-CoV-2) can also be transmitted through nonporous materials such as glass, plastic, and steel, as well as through porous materials such as cloth. Air humidity, sunlight, and the rate of surface absorption affect the survival time of enveloped virus (SARS-CoV-2) in porous and nonporous substances, as discussed below.

## Methods

The SARS coronavirus pathogen does not always stay in the respiratory system once it is established in the human body. For example, some important ways that enveloped virus (SARS-CoV-2) can infect wastewater and groundwater are via patient feces, open-air landfills, and contaminated materials that are left in the environment during rainfall; these contamination routes are crucial to understanding COVID-19 diffusion (Islam 2020).

**Fig. 4:**
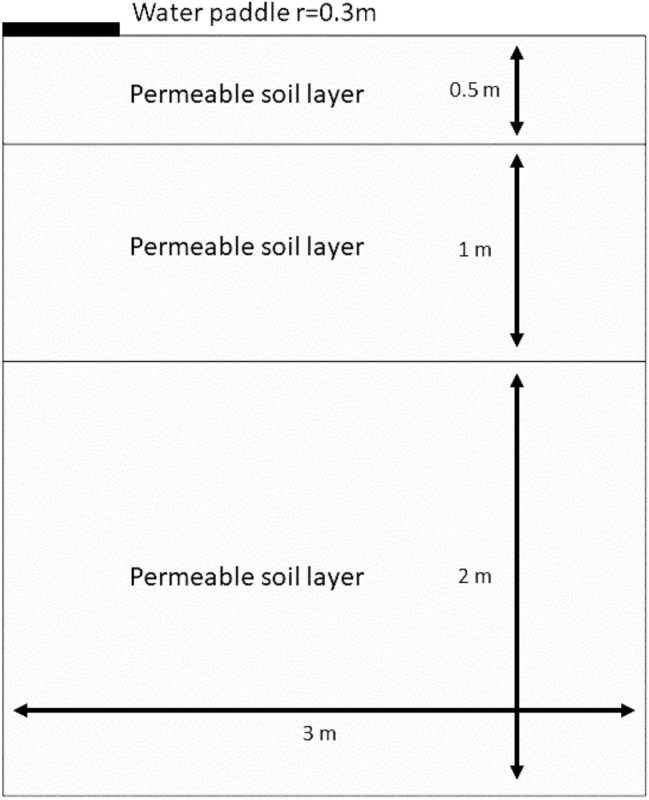
Geometry of the puddle ring and the permeable soil layers that were utilized to model virus diffusion through a layered soil sample

To predict the likelihood of SARS-CoV-2 dispersion into soil through virus-contaminated water, a model of virus diffusion through a layered soil sample was implemented. In this model, water is ponded by a ring on the soil surface. Enveloped virus (SARS-CoV-2) is present in the water puddle, and is transported by the water through the dry soil (Fig. 4). The soil is represented by three layers. The top layer is slightly less permeable than those below. The water moves from the bottom of the ring into the soil. The water level in the ring is known, as is the initial distribution of pressure heads in the soil. There is no flow through the vertical walls or the air–soil interface. The porosities (given as volume fractions) of the three layers were considered to be 0.285, 0.348, and 0.403, respectively, starting from the bottom. The soil was considered to be virus-free initially. Enveloped virus (SARS-CoV-2) moves with the water from the pond into the soil at a constant concentration. The model assumes that the vertical axis through the center of the puddle is a line of 2D symmetry. Solutes can freely leave the soil column with the fluid flow through the other boundaries. This problem was modeled and the solute transport was tracked for 20 days.

## Results and discussion: transport through porous media and mathematical modeling development

The Richards equation governs the saturated–unsaturated flow of water in soil. The soil pores are connected to the atmosphere, so it can be assumed that pressure changes in the air do not affect the flow, meaning that the Richards equation is applicable (Oran and Topol 2020). In terms of the pressure head, the Richards equation reads

1 $$\left(C+\mathrm{Se}S\right)\frac{\partial {H}_{\mathrm{p}}}{\partial t}+\nabla \cdot \left(-K\nabla \left({H}_{\mathrm{p}}+D\right)\right)=0,$$

where *C* denotes the specific moisture capacity (m^−1^); Se is the effective saturation of the soil (dimensionless); *S* is a storage coefficient (m^−1^), which is expressed in terms of* θ*_s_ and* θ*_r_: the volume fractions of fluid at saturation and after drainage, respectively; *H*_p_ is the pressure head (m), which is proportional to the dependent variable *p* (Pa); *t* is the elapsed time; *K* is the hydraulic conductivity (m/s); and *D* (m) is the vertical diffusion depth typically, represent in the *z*-direction and "r" is water paddle radius.

The most general form of the governing equation for virus transport, which considers the convection and diffusion of a sorbing species in variably saturated soil, can be written as follows:

2 $$\frac{\partial }{\partial t}\left(\theta c\right)+\frac{\partial }{\partial t}\left({\rho }_{\mathrm{b}}{c}_{\mathrm{p}}\right)+{\varvec{u}}\cdot \nabla c+\nabla \cdot (-\theta {D}_{L}\nabla c)=0,$$

where *c* is enveloped virus (SARS-CoV-2) concentration (PFU/mL); *t* is the elapsed time; and *c*_p_ is the mass of contaminant adsorbed per dry unit weight of solid (mg/kg). In addition, *ρ*_b_ is the bulk density (kg/m^3^) and *θ* is the volume fraction (porosity) of fluid. Thus, the term *ρ*_b_*c*_p_ is the mass of contaminant attached to the soil. It is worth noting that reaction rate terms were omitted from this preliminary model.

All of the physical and transport proprieties of soil were fixed in accordance with their literature values (Oran and Topol 2020). Enveloped virus (SARS-CoV-2) concentration in the water puddle was considered to be 1 PFU/mL, and the diffusion coefficient of the liquid phase was 3.74 × 10^−3^ m^2^/day. Other physical and transport properties are not reported here for the sake of brevity. Different upper soil layer porosities were also analyzed.

The finite element (FEM) method was used when performing the modeling, which was implemented in COMSOL Multiphysics 5.5. The three soil layers were discretized into a total of 7098 mapped mesh elements with an average element quality of about 0.9993, leading to 35,999 degrees of freedom. The mesh used provided adequate spatial resolution for the system under study. The solution was independent of the grid size, even with further refinements. On an i7-10750 hexa-core processor computer (16 GB DDR4 RAM) running under Windows 10, a typical parametric sweep simulation was completed in about 36 min.

**Fig. 5:**
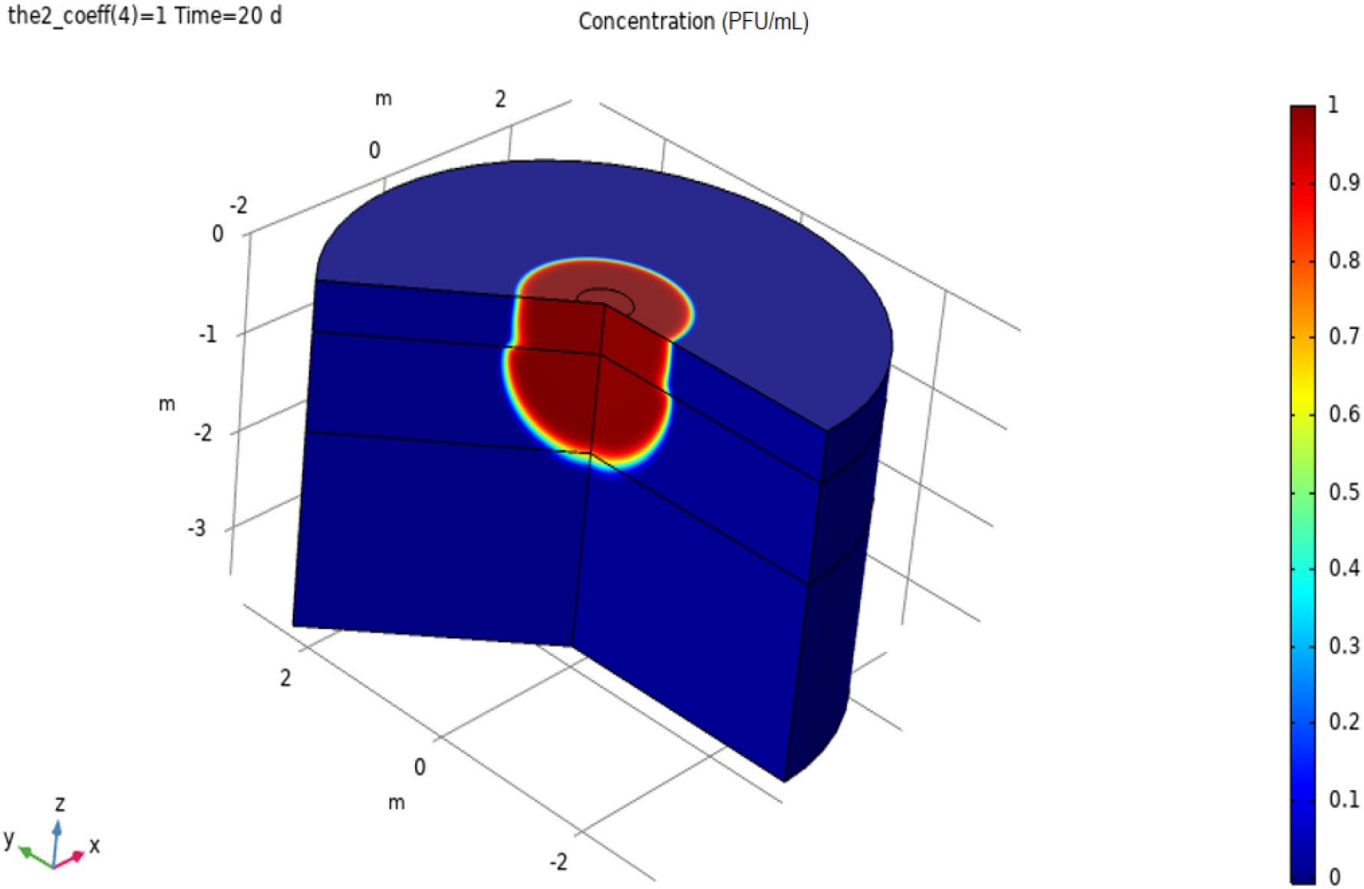
3D model of polluted water transport through porous media

To aid understanding of the model simulation, Fig. 5 presents the diffusion of the polluted water into the 3D model constructed through 3D revolution of the corresponding vertically sliced soil.

**Fig. 6:**
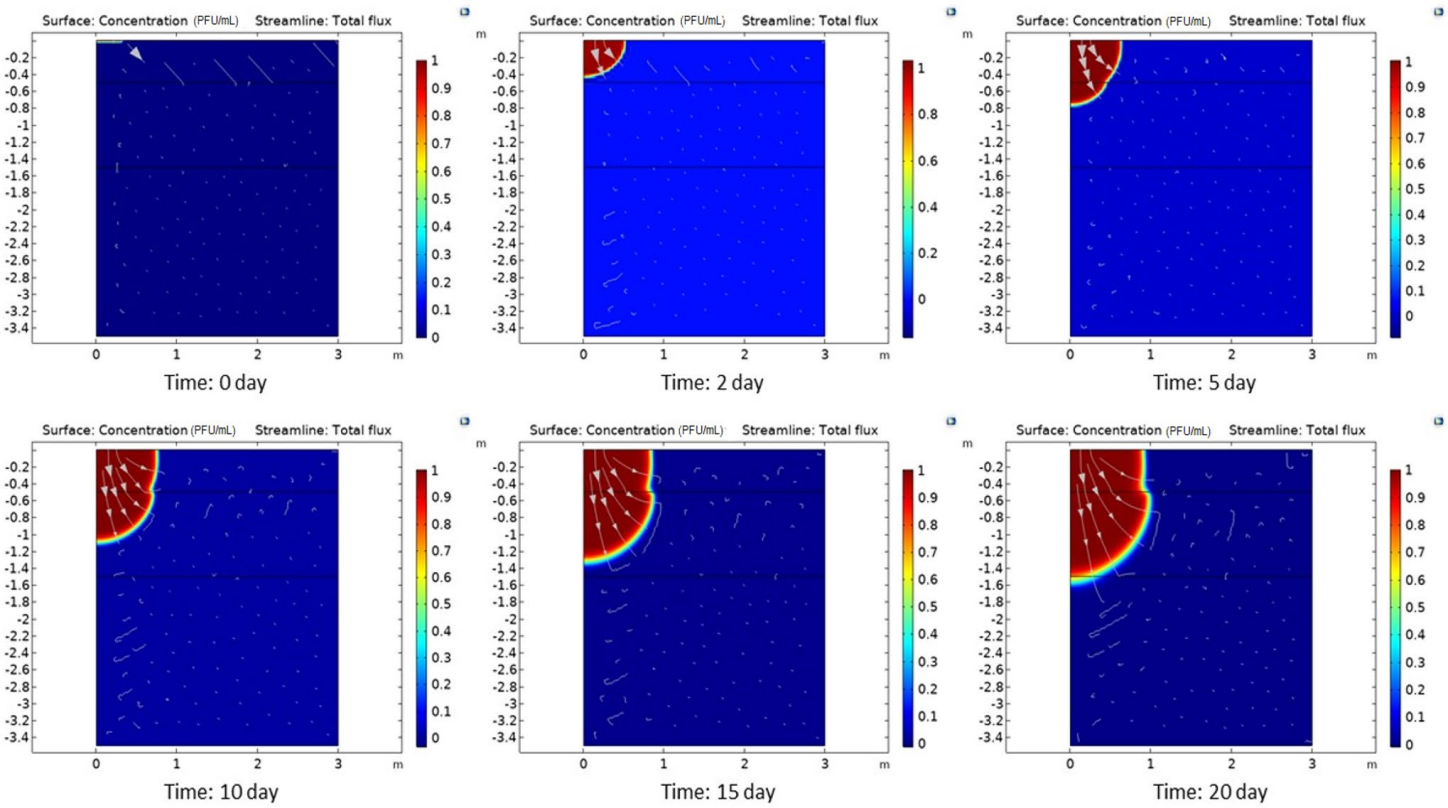
Distribution and streamlines of coronavirus at different times in the three soil sections

Plots showing the time evolution of the distribution of enveloped virus (SARS-CoV-2) considered in 3 soil sections at 0, 2, 5, 10, 15, and 20 days are reported in Fig. 6. In the plots, enveloped virus (SARS-CoV-2) concentrations (in PFU/mL) are normalized to enveloped virus (SARS-CoV-2) concentration in the water pond. Progressive contamination of an increasing proportion of the soil in the second section was observed over the course of 60 h. It is worth mentioning that, in its present form, the model does not consider the decay in virus activity (which could be expressed, for instance, in terms of a decay rate) over time. However, that can easily be included in the model when more detailed information on SARS-COV-2 activity becomes available.

**Fig. 7:**
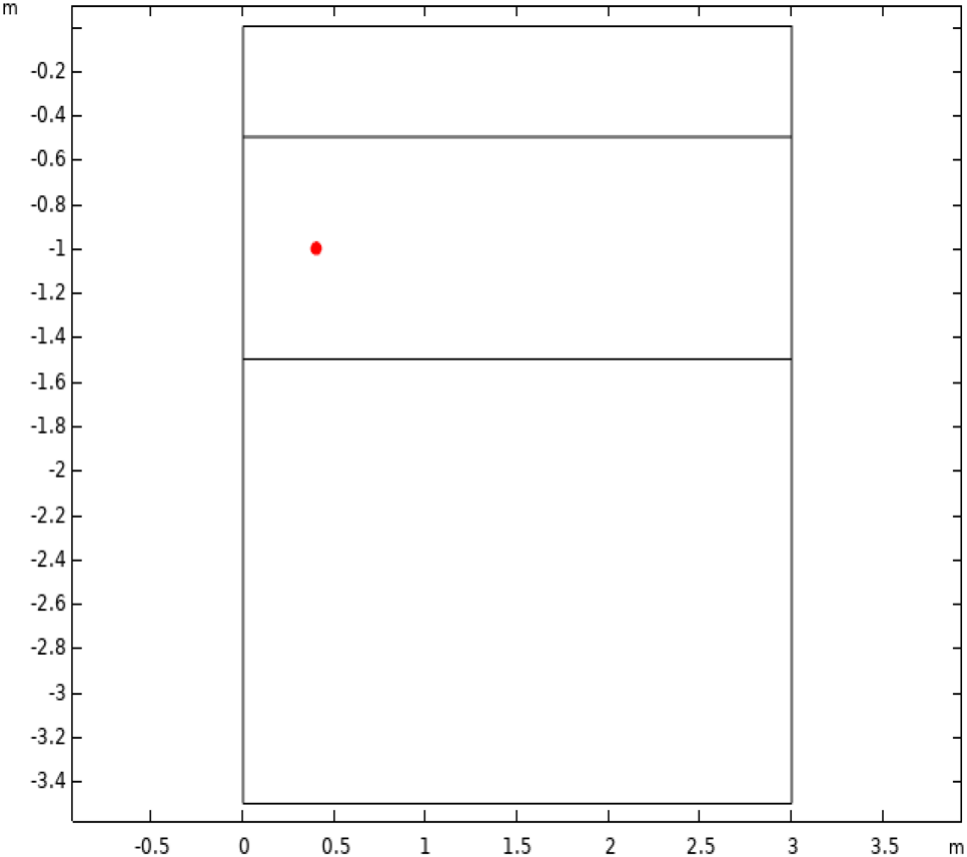
Position of the probe point in the middle layer

**Fig. 8:**
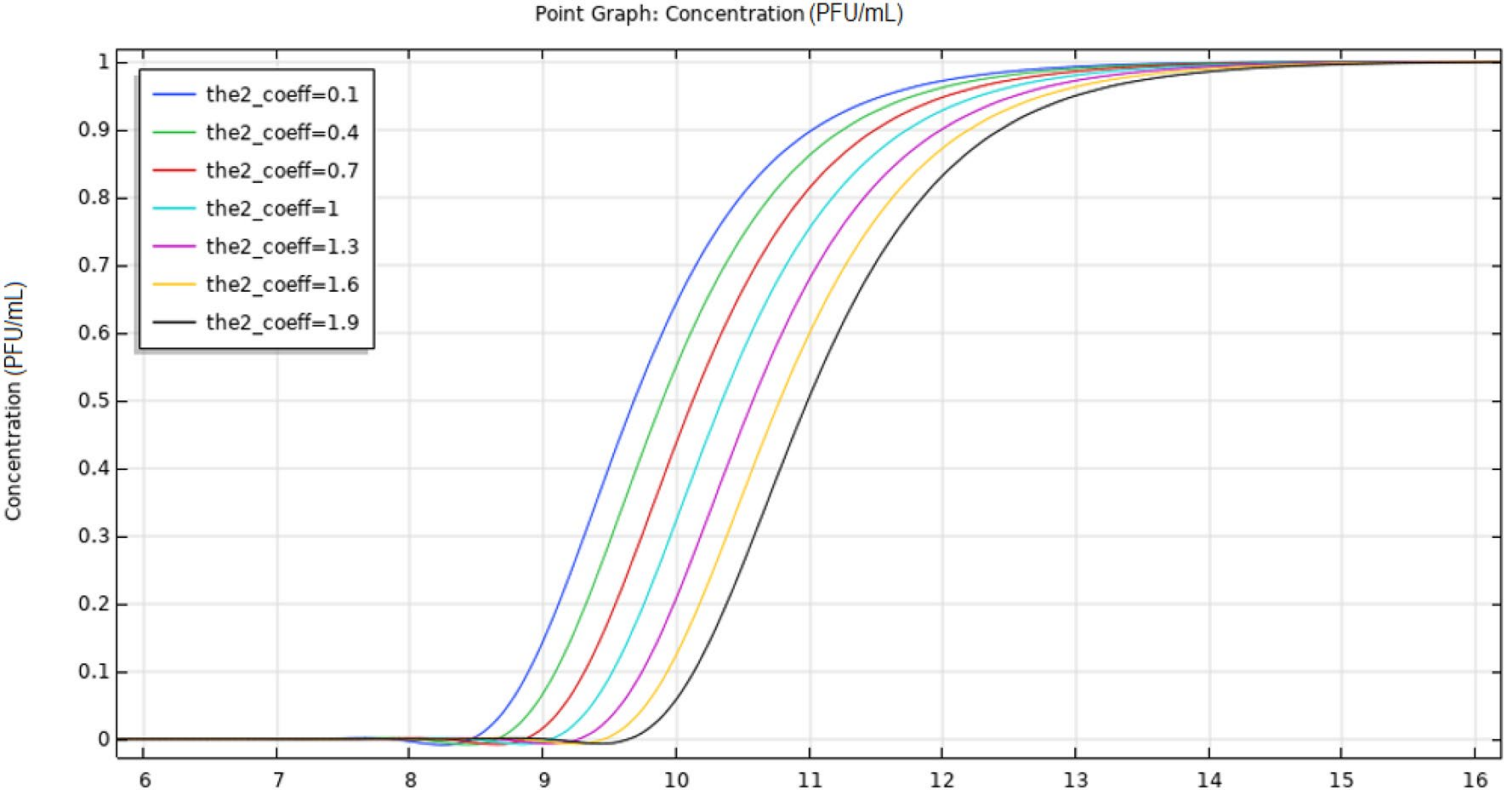
Temporal evolution of enveloped virus (SARS-CoV-2) concentration at the probe point as a function of the upper layer porosity coefficient

The evolution of the coronavirus contamination of the soil and the evolution of enveloped virus (SARS-CoV-2) transport streamlines were examined. The third layer (section) only became contaminated after a long period. Additionally, the influence of the upper layer porosity was investigated by performing a parametric sweep analysis using a coefficient ranging from 10% to 190% of the original porosity. The temporal variation in enveloped virus (SARS-CoV-2) concentration at a probe point in the middle layer was derived for various upper layer porosities. The position of this probe point (depth = − 1 m, *r* = 0.4 m) is shown in Fig. 7, and plots of the parametric concentration at that point over time for different upper layer porosities are reported in Fig. 8.

**Fig. 9:**
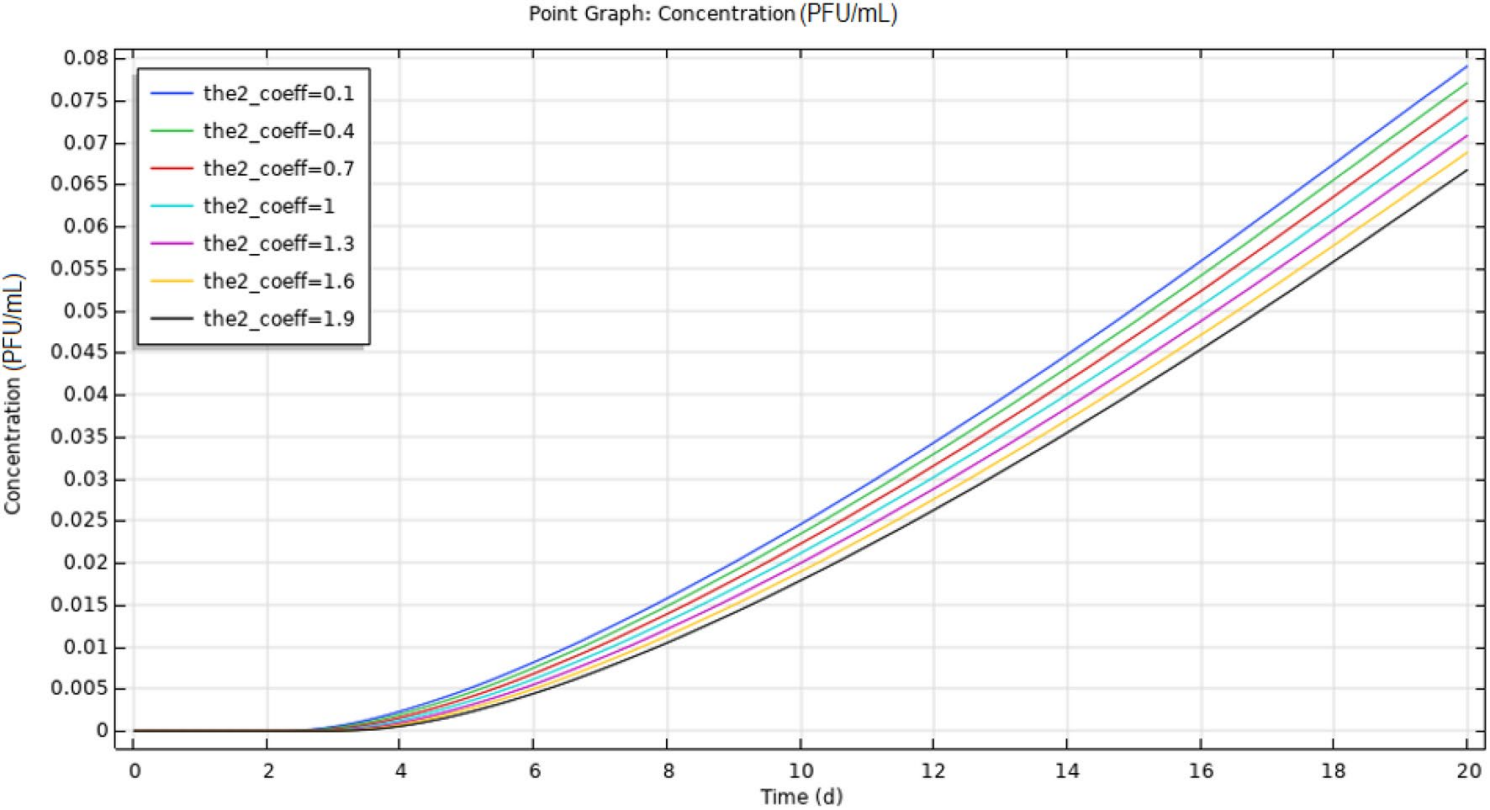
Temporal evolution of the average virus concentration in the middle layer at *r* < 3 m as a function of the upper layer porosity coefficient

The parametric graphs show that contamination is retarded as the upper layer porosity is increased. This suggests that the flow is mostly influenced by the frictional resistance within the pores and that the pressure gradient is the major driving force. The contamination of the middle layer at a depth of 1 m after 8 days was analyzed. The average concentration in the middle layer at *r* < 3 m was also investigated, and is reported in Fig. 9.

In a limited area of the selected soil section, the contamination was seen to be limited to a maximum of 0.1 PFU/mL after 20 days of contaminated water drainage.

It should be noted that the results presented for this model are from a preliminary investigation. More detailed information on the decay rate of enveloped virus (SARS-CoV-2), detailed diffusion data, and other physical quantities will be updated and included in the model as soon as more information on the SARS-CoV-2 virus is available in the literature. Also, we considered that enveloped virus (SARS-CoV-2)-contaminated water in our model was tap water or fresh water, not wastewater. Wastewater contains residual wastes that could affect the diffusion of enveloped virus (SARS-CoV-2) into the soil. To account for the effects of waterwater components on virus diffusion through the soil in our model, we require more detailed information on the physical and/or chemical interaction mechanisms between enveloped virus (SARS-CoV-2) and other water contaminants to be published in the literature.

## Conclusions

In this brief review and investigation, we have highlighted the risks of uncontrolled personal protective equipment disposal and the mechanism of virus transmission from contaminated surfaces to humans and groundwater. Considering the risk factors involved, PPE should not be reused without proper disinfection. PPE should be worn and removed according to the recommendations of the CDC and WHO, as this should reduce the risk of cross-contamination. PPE should be changed and disinfected at regular intervals (after about 8 h of continuous utilization). Due to the shortage of PPE caused by the current COVID-19 pandemic, there is a strong drive around the world to reuse PPE. However, if the reused PPE is not properly disinfected, enveloped viruses such as SARS-CoV-2 can spread across its surface. All medical wastes, including defective PPE components, should be discarded in designated appropriate containers and disposed of in landfills. It is also known that viruses can diffuse through porous soil, eventually contaminating groundwater. Proper treatment of enveloped viruses is absolutely necessary to reduce virus transmission and contamination of groundwater sources, even in arid areas. The agencies responsible for the proper disposal of used PPE must ensure that this waste material does not pollute the environment. Enveloped virus (SARS-CoV-2) transport model presented in this paper can easily be applied to other enveloped viruses if relevant chemical and physical transport properties are known. We have presented only the main results of our model and simulations here. More analytical research is necessary to determine all of the causes of infections from enveloped viruses and their transmission pathways, as this should enable the creation of adequate guidelines and safety protocols that will prevent their spread and effectively control infections.
